# NEIGHBORHOOD HEALTH CARE FACILITIES AND INCIDENT DEMENTIA: RESULTS FROM THE EINSTEIN AGING STUDY

**DOI:** 10.1093/geroni/igad104.3494

**Published:** 2023-12-21

**Authors:** Jinshil Hyun, Charles Hall, Mindy Katz, Carol Derby, Richard Lipton

**Affiliations:** Albert Einstein College of Medicine, Bronx, New York, United States; Albert Einstein College of Medicine, Bronx, New York, United States; Albert Einstein College of Medicine, Bronx, New York, United States; Albert Einstein College of Medicine, Bronx, New York, United States; Albert Einstein College of Medicine, Bronx, New York, United States

## Abstract

Though access to health care is associated with improved chronic health outcomes, data on dementia risk are limited. From the Ecological Model of Aging, limited access to health care facilities may be associated with increased likelihood of developing dementia through poor management of comorbidities. The current study aims to investigate the association between the density of neighborhood health care facilities and incident dementia. Participants were community-dwelling older adults, systematically recruited from Bronx, NY (N=1,461, mean age=78.4; mean follow-ups=4.6 years; 68% non-Hispanic White, 26% non-Hispanic Black). Density of health care facilities in each participant’s Census Tract was derived from the National Establishment Time-Series data. We used Cox proportional hazards models using age as the time scale and adjusted for sex, race, education, income, and neighborhood deprivation. Incident dementia developed in 10% of the sample. Older adults living in the areas with lower density of health care facilities, compared to those living areas with a higher density of health care facilities, were at increased risk for incident dementia (HR=1.79, 95% CI=[1.12, 2.87] for lowest vs. highest tertile; HR=1.65, 95% CI=[1.02, 2.66] for intermediate vs. highest tertile). Further controlling for cardiovascular and other comorbidities did not change the pattern of results. The current results suggest that unequal distribution of health care facilities across geographic locations may help account for cognitive health disparities among older adults. Future studies will need to identify behavioral and individual-level mechanisms (e.g., healthcare use, better treatment before disease occurs) in the association between neighborhood health care facilities and dementia.

